# Incorporating familial risk, lifestyle factors, and pharmacogenomic insights into personalized noncommunicable disease (NCD) reports for healthcare funder beneficiaries participating in the Open Genome Project

**DOI:** 10.1111/ahg.12582

**Published:** 2024-10-29

**Authors:** Manie De Klerk, Nicole Van Der Merwe, Johny Erasmus, Lindiwe Whati, Kelebogile E. Moremi, Daniel W. Olivier, Maritha J. Kotze

**Affiliations:** ^1^ Health Care Leadership University of Stellenbosch Business School Cape Town South Africa; ^2^ Division of Chemical Pathology, Department of Pathology, Faculty of Medicine and Health Sciences Stellenbosch University Cape Town South Africa; ^3^ FamGen Counselling Bloemfontein South Africa; ^4^ Momentum Group Cape Town South Africa; ^5^ Gknowmix (Pty) Ltd. Cape Town South Africa; ^6^ Division of Chemical Pathology, Department of Pathology, National Health Laboratory Service Tygerberg Hospital Cape Town South Africa

**Keywords:** benefit‐sharing, general practice, genetic counseling, genomic medicine, learning, medical scheme, open innovation, pathology‐supported genetic testing

## Abstract

**Introduction:**

An ethics‐guided decision‐making framework was developed for applying pathology‐supported genetic testing, a multifaceted pharmacodiagnostic approach that translates population risk stratification into clinical utility. We introduce this service, supported by the Open Genome Project, which aligns with the beneficence principle in personalized medicine.

**Methods:**

Genetic testing of six noncommunicable disease pathways was conducted as part of a pilot program, benchmarked against a readiness checklist for implementation of genomic medicine in Africa. Patient referral criteria were determined using healthcare funder claims data, employing the Adjusted Clinical Groupers and Resource Utilization Band risk rating structure to identify potential nonresponders to treatment.

**Results:**

Three of the 135 doctors (2.2%) invited expressed immediate disinterest in the pilot, while 24 (17.8%) actively participated. Inherited, lifestyle‐triggered, and therapy‐related pathologies were simultaneously assessed in case reports, with special medical scheme reimbursement tariff codes applied to 25 patient referrals. The findings were used by the participating genetic counselor to select three patients for whole exome sequencing, utilizing a novel, level‐up data processing algorithm for adaptive reporting.

**Conclusion:**

This study demonstrated the implementation of genomics into an evolving workflow for patients with a history of frequent clinic visits. Eliminating the cost barrier provided valuable insights to guide future reimbursement policy decisions.

## 1 INTRODUCTION

The looming economic crisis posed by noncommunicable diseases (NCDs) in South Africa, considered from a global perspective (Samodien et al., 2021), increased the interest in genomic medicine as a healthcare solution for Africa harboring significant genetic diversity (Tishkoff et al., 2009). Although it is well known that interaction of environmental exposures with the human genome could explain interindividual differences in treatment outcome of major NCDs such as cancer, cardiovascular disease (CVD), and mental health disorders, application of this knowledge base is limited by dissemination gaps. The four‐step clinical implementation process outlined by Khoury et al. (2007), for the evaluation of analytic and clinical validity, clinical utility, and ethical, legal, and social implications (ACCE) of genetic tests, has limited applicability in the realm of public health genomics (Cleeren et al., 2011). The potential risks and benefits of integrating genome‐based knowledge and technologies into health services and public policy require careful consideration, as the ethical implications and clinical impact of genomics can affect nearly every medical condition (Johnson et al., 2020; Jongeneel et al., 2022). For prevention and optimal treatment of NCDs with complex multifactorial inheritance, a modified approach compared to that used for rare single‐gene disorders has therefore been suggested by Kotze (2016). This relates to the shift from genetics focused on single‐gene Mendelian inherited traits, toward genomics for assessment of the broader effect of multiple interacting genes and their interplay with environmental factors.

The pursuit of a new approach to managing NCDs as a group of disorders characterized by both distinct and shared disease pathways, stems from recognizing the limitations of stand‐alone laboratory tests and the realization that genetic information may be insufficient to predict response to treatment. The benefit of genomic testing to refine risk stratification and identify individuals with different treatment requirements has been demonstrated in numerous studies performed in South Africa and elsewhere (Johansson et al., 2023; Marais et al., 2019). A single genetic risk factor, even as severe as heterozygous familial hypercholesterolemia (FH), does not directly cause a heart attack. This insight emerged from germline DNA testing using the polymerase chain reaction (PCR) by Kotze et al. (1989) for the first time in South Africa. Delineation of the molecular basis of FH characterized by a founder gene effect (Kotze et al., 1991), enabled cost‐effective diagnostic, prenatal, and presymptomatic DNA screening (Kotze et al., 1998; Vergotine et al., 2001). Newly developed methodologies were validated in adult and pediatric population groups, both within South Africa and across the world, to provide the foundational basis for an interdisciplinary risk management strategy for relatively common, preventable genetic disorders (Kotze & Callis, 1999). Identification of overlapping disease pathways led to the incorporation of the FH test into a multigene CVD strip‐assay (Kotze et al., 2003; Kotze & Thiart, 2003), which evolved into a dynamic pathology‐supported genetic testing (PSGT) platform underpinned by genomic applications for a wide range of NCDs. These include Alzheimer's disease (Kotze & Van Rensburg, 2012; Lückhoff et al., 2016), breast cancer (Mampunye et al., 2021; Okunola et al., 2023), hereditary hemochromatosis (HH; Kotze et al., 2004, 2009), major depressive disorder (MDD; Delport et al., 2014; Van Der Merwe et al., 2012), and multiple sclerosis (Johannes et al., 2023; Kotze et al., 2006). PSGT incorporating multigene testing based on the scientific evidence provided in these studies has bridged the clinical implementation gap between limited first‐tier genotyping and comprehensive genomic sequencing (Baatjes et al., 2023; Marais et al., 2019; Van der Merwe et al., 2017; Van Rensburg et al., 2019).

NCDs exert a widespread influence on healthcare, ranging from the promotion of wellness to the management of illness. This paradigm introduced new ethical duties for researchers who collect and share data to advance personalized genomic medicine (Vos et al., 2017). In this context, Kotze et al. (2024) developed an ethics framework shaped by the challenges encountered during the implementation of precision oncology in South Africa. Inadequate coordination of care among healthcare practitioners, arising from fragmented clinical and laboratory services, has surfaced as a significant implementation barrier and ethical dilemma that extends across health disciplines. This predicament leaves patients in a state of uncertainty regarding whom to approach for inquiries or concerns, resulting in delayed access to information when they need it most. To address this challenge, we implemented a flexible workflow under the Open Genome Project. This initiative collects patient data for generation of adaptable feedback reports, provided to clinicians in the form of individualized case presentations with three action points based on the likelihood of inherited, lifestyle‐triggered, and therapy‐induced pathologies. By synthesizing this wealth of knowledge in a transdisciplinary manner (Garton et al., 2022), the delivery of patient‐centered care can be provided within a robust measurement framework for personalized health interventions. Additionally, collaborative knowledge generation enables continual process improvement to uphold ethical values that increase the benefit to participants, researchers, and funders alike. In this context, Christowitz et al. (2024) provided several examples of PSGT applicable to breast cancer, which highlighted three novel aspects aimed at fostering diverse perspectives across clinical domains. These innovations are centered around a unique computational process for adaptive reporting, enhanced by genomic counseling support. Each step builds upon the previous one:

1. A mobile phone app and wellness survey aid in risk stratification, using the first‐level data processing algorithm.
2. Rapid point‐of‐care (POC) genotyping, combined with pathology data, increases access to the appropriate care pathway, utilizing the second‐level data processing algorithm.
3. Extended genetic testing with disease‐specific microarray and next‐generation sequencing (NGS) options provides a comprehensive patient report, leveraging the third‐level data processing algorithm.

The PSGT infrastructure was harnessed in this study with the aim to develop a new medical scheme reimbursement model for NCDs with an intervening genetic component. The first objective was to successfully enroll healthcare funder beneficiaries into a pilot access program using medical claims data. The second objective involved first‐tier genetic testing across multiple NCD pathways as a screening step to introduce comprehensive NGS using whole exome sequencing (WES) into clinical practice. Preauthorization by the healthcare funder demonstrated the real‐world applicability of PSGT in identifying patients and financing genome sequencing for clinical use, rather than solely for research. Although the emergence of the coronavirus disease in 2019 (COVID‐19) disrupted sample collection and patient consultations, it underscored the importance of routine biochemistry biomarkers utilized in clinical practice for identifying genetic underpinnings of the metabolic syndrome as a potential outcome of prolonged symptoms (Menezes et al., 2023). Our findings provide a real‐world example of integrating data‐driven discoveries and patient experiences with external scientific evidence to put new knowledge into practice.

## 2 MATERIALS AND METHODS

### 2.1 Ethics approval

This study was approved with annual review by the Human Research Ethics Committee of Stellenbosch University (N09/08/224). All participants provided written informed consent, either on paper or online, as approved for the Open Genome Project (C19/06/020). While extensive self‐citation was necessary due to the relevance of prior work, it was conducted with transparency and ethics approval of this article under reference number HREC2‐2024‐7545.

### 2.2 Study design

**FIGURE 1:**
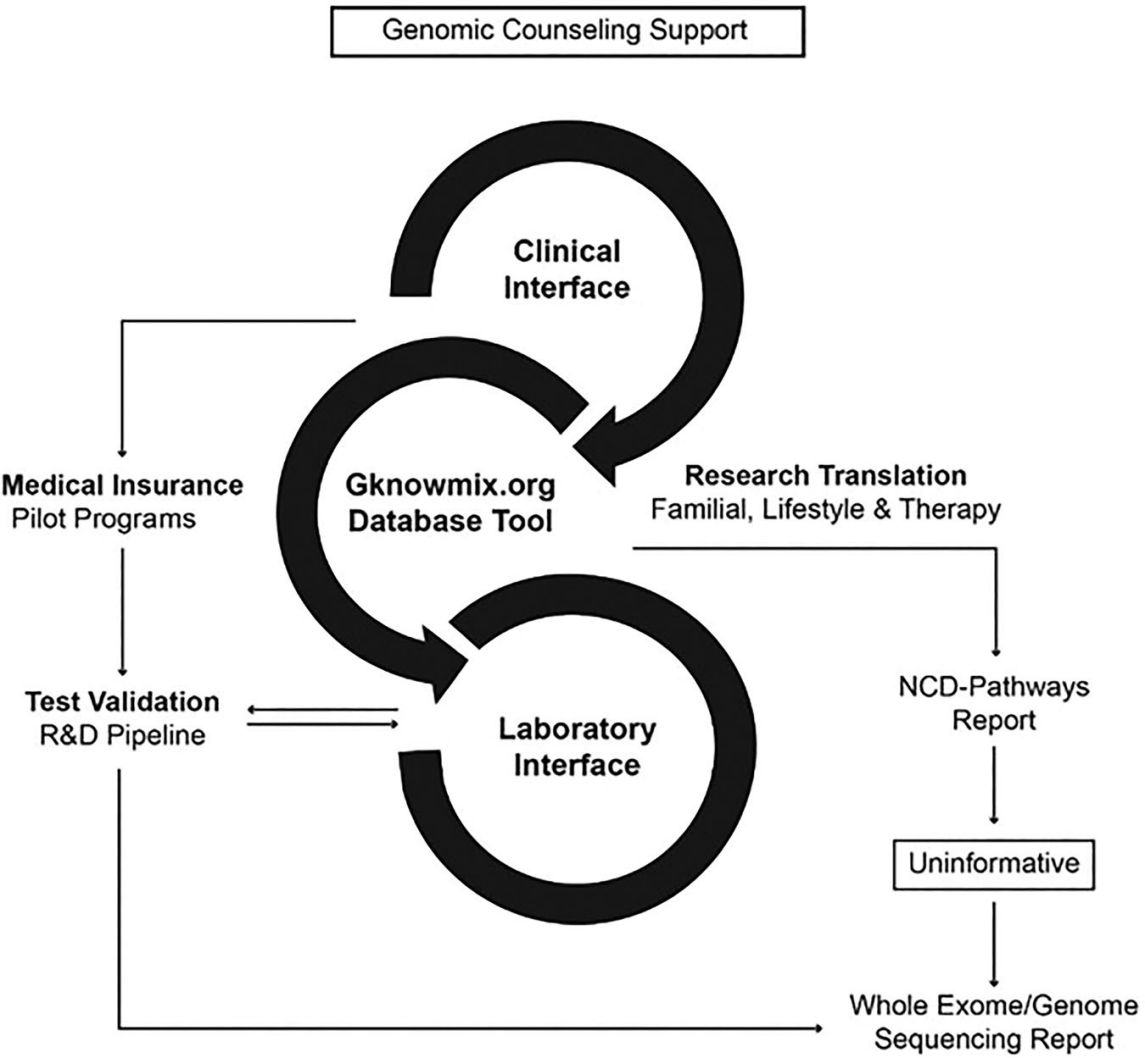
Pathology‐supported genetic testing strategy for development of a new medical scheme reimbursement model for noncommunicable diseases (NCDs) with an intervening genetic component. A three‐pronged approach is used toward identification of (1) pathogenic variants associated with familial risk, (2) genetic underpinnings of disease/tumor subtypes, biochemical abnormalities, or comorbidities influenced by modifiable environmental or lifestyle risk factors, and (3) patients at increased risk of medication side effects or failure due to genetic variation evaluated in different clinical contexts (adopted with minor modification from Accelerating Excellence in Science in Africa (AESA), 2021).

A mixed‐methods approach employing both qualitative and quantitative data collection through an embedded sequential exploratory study designed for the Open Genome Project was implemented to enable the research translation process illustrated in Figure 1. The Readiness Checklist (RC) published by Jongeneel et al. (2022) was used to assess the feasibility of extending the NCD pathway analysis to WES based on the available infrastructures in South Africa for implementing a combined chronic disease and wellness screening program. The framework underpinned by the RC served as a benchmark for identifying implementation gaps encountered during research translation. The collaborative PSGT process involves the generation of a personalized NCD pathways report for each patient, to identify immediate action points and to indicate the need for extended testing using WES in uninformative cases. For this purpose, four steps were used to structure the different data sets into familial, lifestyle, and therapy‐associated risk categories via a reporting algorithm that integrates research with service delivery:

1. *Research*: Commonalities exemplified by metabolic syndrome features relevant across the NCD spectrum were identified and included in a prescreening assessment. This set the context for genetic testing based on each patient's clinical characteristics, collected through a wellness survey (study questionnaire).
2. *Data integration*: Patient genotypes were correlated with relevant biochemical findings from previous pathological assessments when available. The personal medical history, family history, lifestyle risk scores, and treatment response information gathered in step 1 were used for clinical interpretation as part of test validation.
3. *Service*: Clinical interpretation of the genetic data was supported by medical information provided by referring clinicians and patients, such as body mass index (BMI) and medication use. This helped categorize risks related to familial, lifestyle, and therapy‐associated pathologies.
4. *Data reuse*: In cases where initial tests were uninformative, extended testing using WES was performed. This step applied a unique level‐up data processing algorithm designed for adaptive reporting and patient follow‐up.

The above‐mentioned PSGT process involves transferring assays from laboratory‐based instruments to a portable POC device (ParaDNA^®^) using multiplex HyBeacon^®^ probe technology as part of skills training in analytical validation. This approach recognizes that many chronic diseases share common genetic and lifestyle factors. In addition to highlighting the most relevant information for each patient on the first page of the reports directed to the referring general practitioners (GPs), first‐tier genotyping also served as a quality control step through cross‐referencing with overlapping WES data covering the coding regions of all human genes. Ongoing comparative effectiveness studies support accurate sample and data management, especially when high‐throughput reference laboratories are involved in returning research results.

### 2.3 Patient enrolment based on healthcare funder claims data

The Adjusted Clinical Groupers and Resource Utilization Band (ACG RUB) structure developed by Johns Hopkins University (https://www.hopkinsacg.org/wp‐content/uploads/2022/08/Johns‐Hopkins‐ACG‐System_Overview‐Brochure‐v080222.pdf) was adopted by the participating medical scheme. The program family physician (PFP) used this system to identify healthcare funder beneficiaries in the four highest risk ACG RUB Groups in 2018 and 2019. The patient inclusion criteria were based on frequent appointments with a GP for medical conditions where a lack of early intervention is associated with poor clinical outcomes and increased cost to the medical scheme. These include hypercholesterolemia and associated medical conditions such as CVD and dementia/Alzheimer's disease, cancer, depression, deep vein thrombosis, pulmonary embolism, recurrent pregnancy loss, iron deficiency or overload/hemochromatosis, and/or adverse treatment response relating to these medical conditions. Exclusion of patients with none of these NCDs resulted in a total of 258 patients considered suitable for potential participation in the pilot program. The treating GPs were contacted between January 2019 and June 2021 by the PFP appointed by the healthcare funder as part of their internal implementation process. Of 247 calls made by the PFP, 135 GPs representing 148 patients were successfully reached by telephone and informed of the initiative, followed by an email with more detailed information on the pilot program. Clinicians who referred patients for PSGT, after obtaining informed consent and administering the study questionnaire, used specific claim codes for genomic consultations. The study questionnaire, included with the saliva sample collection kits used to obtain patient samples at various GP practices between August 2019 and September 2022, helped identify lifestyle factors relevant to the genes tested.

### 2.4 Genetic testing of NCD pathways across six metabolic pathways

Genetic testing was conducted at the Pathology Research Facility, Faculty of Medicine and Health Sciences, Stellenbosch University. DNA was extracted from saliva samples with Oragene reagents according to the manufacturer's instructions, and genotyped using real‐time PCR‐technology. Predesigned TaqMan^®^ SNP Genotyping Assays were used according to the Thermo Fisher Scientific User Guide (https://www.thermofisher.com/za/en/home/life‐science/pcr/real‐time‐pcr/real‐time‐pcr‐assays/snp‐genotyping‐taqman‐assays/single‐tube‐snp‐genotyping.html), on the Roche LightCycler^®^ 480 II. Genotypes generated for the single nucleotide polymorphisms (SNPs) listed below (assay IDs highlighted in brackets) were integrated into a unique NCD pathways report for each patient, based on the scientific evidence for the clinical interpretation matrix outlined in Table 1, as previously conceptualized by Kotze (2016). The selected SNPs display pleiotropic effects across at least six metabolic pathways influenced by inherited and environmental risk factors shared by many NCDs, as the starting point for introduction of WES across an unlimited number of genes and variants in uninformative cases studied. Replication studies conducted in South Africa highlighted the clinical implications for multiple traits relating to the study inclusion criteria:

1. *Apolipoprotein E* (*APOE*) *rs7412* (**C_904973_10**)*, rs429358* (**C_3084793_20**): These variants are important for differentiating FH that requires cholesterol‐lowering treatment to prevent premature heart attacks, from hyperlipoproteinemia type III and milder forms of dyslipidemia associated with late‐onset Alzheimer's disease and metabolic syndrome. These conditions can be managed effectively or prevented with targeted lifestyle changes, which should be implemented at diagnosis aided by genetic testing to avoid overtreatment.
2. *Methylenetetrahydrofolate reductase* (*MTHFR*) *rs1801133* (**C_1202883_20**)*, rs1801131* (**C_850486_20**): Highlights the importance of a balanced B‐vitamin intake above the recommended daily dose to prevent or restore dysfunction of the methylation pathway when folate status is low. Continuous availability of the nutrient cofactors required for optimal MTHFR enzyme function increases the efficacy and reduces the side effects of certain medications, and helps prevent or address pregnancy complications, cancer, depression, hypertension, and many other medical conditions correlating with hyperhomocysteinemia as a marker for vitamin B12 and/or folate deficiency.
3. *Factor II/prothrombin* *rs1799963* (**C_8726802_20**)*, Factor V* (*FV*) *rs6025* (**C_11975250_10**): Indicates the need to consider inherited thrombophilia in cases of unexplained recurrent pregnancy loss, deep vein thrombosis, and pulmonary embolism. These conditions may be exacerbated by environmental factors such as smoking, immobility, or the use of oral contraceptives or hormone therapy, which may be contraindicated in genetically predisposed individuals.
4. *Hereditary hemochromatosis* (*HFE*) *rs1799945* (**C_1085600_10**), *rs1800562* (**C_1085595_10**): Aids in the differential diagnosis of HH, a preventable iron overload genetic disorder, and insulin resistance hepatic iron overload syndrome, also known as dysmetabolic iron overload as a common cause of hyperferritinemia. This distinction is crucial to avoid invasive liver biopsies and to determine the need for regular phlebotomy or blood donation, while avoiding iron supplementation. Managing iron status through regular monitoring of relevant blood biochemistry and avoidance of excessive alcohol consumption and other environmental risk factors is crucial to prevent organ damage.
5. *Transmembrane protease, serine 6* (*TMPRSS6*) *rs855791* (**C_3152883_20**): Assists in differentiating between familial iron deficiency and anemia of chronic disease. These conditions are often linked to inflammation and/or obesity which are key features of the metabolic syndrome that contributes to COVID‐19 severity. This distinction may require extended nutrigenetic testing of additional SNPs performed on a case‐by‐case basis, to assess potential gene–diet interactions. Iron is essential for oligodendrocytes in the central nervous system to synthesize myelin sheaths. Iron dysregulation is particularly relevant to neurodegenerative diseases linked to vitamin D deficiency due to the effect on hepcidin production mediating iron availability for erythropoiesis and oxygen transport.
6. *Cytochrome P450 family 2 D member 6* (*CYP2D6*) *rs3892097* (**C_27102431_D0**): Emphasizes the need to consider medication side effects and potential treatment failure with up to 25% of commonly prescribed drugs, including certain statins and antidepressants, as well as tamoxifen for hormone receptor‐positive breast cancer. Certain selective serotonin reuptake inhibitors that inhibit CYP2D6 function pose the highest risk. Therefore, extended pharmacogenetic testing of additional SNPs was conducted on a case‐by‐case basis to assess potential gene–drug and drug–drug interactions. In CYP2D6 poor metabolizers with hereditary breast and ovarian cancer syndrome (HBOC) caused by a pathogenic *BRCA1/2* gene variant, treatment with tamoxifen is associated with treatment failure.

**TABLE 1:** Matrix for data integration and report generation incorporating published data into the pathology‐supported genetic testing platform, extending from first‐tier genotyping to whole exome/genome sequencing in cases where initial results are uninformative.

Disease pathway analysis	Family medical history and genetic susceptibility	Environmental factors and treatment response	Evidence base for PSGT across six pathways
**Clinical risk profile** According to the inclusion criteria	Screening for genetic underpinnings of the metabolic syndrome and variants in genes with pleiotropic effects (*APOE*, *MTHFR*, *FII*, *FV*, *HFE*, and *TMPRSS6*)^a^ to determine their clinical relevance to the personal and family medical conditions documented for each patient.	Questionnaire‐based assessment of dietary status (based on fat, folate, fruit, and vegetable intake) and other lifestyle factors (physical activity, smoking, and alcohol consumption) known to interact with the genetic variants included in the first‐tier genotyping assay.	Kotze et al., 2005 Kotze et al., 2009 Kotze and Van Rensberg, 2012 Van Der Merwe et al., 2012 Davis et al., 2014 Kotze et al., 2014 Delport et al., 2014 Lückhoff et al., 2016 Kruger et al., 2016 Van Der Merwe et al., 2017 Moremi et al., 2018 Van Rensburg et al., 2019 Baatjes et al., 2023 Okunola et al., 2023
**Pathology test results** Available from previous/requested assessments	Assessment of relevant pathological indicators that may reflect gene–environment interactions as biological intermediates to help determine the need for extended testing of pathogenic variants in high‐ to moderate‐penetrance genes (*BRCA1*/2)^a^ in uninformative cases.	Monitoring of relevant pathology/biochemical test results in relation to intermediate phenotypes such as the metabolic syndrome and medication side effects/failure in the presence or absence of pharmacogenetic targets (*CYP2D6*)^a^.	

Abbreviations: *APOE*, apolipoprotein E; *CYP2D6*, cytochrome P450 family 2D member 6; *FII*, factor II/prothrombin; *FV*, factor V; *HFE*, hereditary hemochromatosis; *MTHFR*, methylenetetrahydrofolate reductase; *PSGT*, pathology‐supported genetic testing.; *TMPRSS6*, transmembrane protease, serine 6.

^a^ The number of genes/variants may be extended on a case‐by‐case basis based on the results presented in Table S1, supplementary to this table. Except for *HFE* and *TMPRSS6*, all the selected gene variants are mentioned in FDA‐approved drug labels (https://www.fda.gov/media/124784/download?attachment).

The clinical characteristics and lifestyle risk scores of healthcare funder beneficiaries were assessed in relation to first‐tier genotyping (Table S1), to identify hyper‐responders to environmental exposures and to highlight combined phenotypic effects that may not be detectable by standard statistical methods (Blanco‐Gómez et al., 2016). This relates to the metabolic syndrome characterized by hypertension, insulin resistance/type II diabetes (fasting glucose ≥5.6 mmol/L), dyslipidemia (elevated triglycerides ≥1.7 mmol/L and/or low high‐density lipoprotein [HDL]‐cholesterol at <1 mmol/L in males and <1.3 mmol/L in females), and central obesity (waist circumference ≥ 94 cm in males and ≥ 80 cm in females), assessed as a unifying risk factor to help distinguish between inherited and lifestyle‐related NCDs. Genetic underpinnings of the metabolic syndrome diagnosed by three or more of these features were evaluated by a registered dietitian (RD) for interpretative nutrigenetic commenting according to the report example published by Daramola et al. (2021), and used for patient follow‐up in the COVID‐19 era. The individualized health guidelines provided were indicated as experimental in the patient reports, given that the success of chronic disease and wellness programs depends on personalized goal‐setting and monitoring, tailored to each patient's unique clinical and genetic risk profile. Established guidelines for genetically guided drug selection or dosing were applied to healthcare funder beneficiaries considering a change of medication, with input from a clinical pharmacologist (CP) when necessary.

### 2.5 Whole exome sequencing and return of research results

The Gknowmix™ software program was used to integrate clinical, pathology, and genetic data into adaptable patient reports, for return of research results applying the ethical principles for WES published by Torrorey‐Sawe et al. (2020). Genotyping on the Roche LightCycler^®^ 480 II was performed in parallel with a portable device (ParaDNA^®^) utilizing multiplex HyBeacon^®^ probe technology. The latter described by French et al. (2001) facilitates rapid first‐tier genotyping with a novel POC test kit (https://gtr.ukri.org/projects?ref=103993) developed to generate automated reports for patients with breast cancer and associated comorbidities (Baatjes et al., 2023). This PSGT approach could be extended to any of the NCDs defined by the study inclusion criteria, as outlined by Kroon et al. (2024) for a patient with familial pulmonary embolism, recurrent pregnancy loss, and warfarin resistance. In this case, WES was preceded by NCD pathway analysis including warfarin dosing POC testing as the pharmacogenetics component. The same WES report format was used in the present study to share risk stratification outcomes, clinical implications, and actionable information with each GP and their patients. The genetic counselor (GC) may use these reports rapidly available through POC testing (ParaDNA^®^), along with age at disease diagnosis and the family history, to select candidates for WES. The Manchester Scoring system was used to determine the likelihood of a *BRCA1/2* pathogenic variant in the family (Evans et al., 2017), together with the CanRisk risk prediction tool to assess the lifetime risk for contralateral breast cancer (Archer et al., 2020; Carver et al., 2021; Lee et al., 2019). The assessment of the metabolic syndrome was part of the WES prescreen algorithm as previously described for breast cancer patients (Van Der Merwe et al., 2012, 2017). For patients in which the genetic and pathology test results as combined in their first‐tier reports were insufficient to explain treatment failure/side effects or a strong family history of the studied NCDs, extended genetic testing was performed using WES in eligible cases.

WES was performed at the Central Analytical Facility of Stellenbosch University, using the Ion AmpliSeq™ Exome RDY kit on the Ion Proton™ sequencing system (Thermo Fisher Scientific), followed by variant calling on the TorrentServer. Variants in preselected genes using virtual WES panels were annotated in the IonReporter and prioritized by using an in‐house pipeline of clinically relevant genes selected from the coding regions of approximately 20,000 human genes (Okunola et al., 2023). Sanger sequencing was used to confirm the detection of pathogenic/likely pathogenic variants identified in any of the cancer susceptibility genes selected for WES analysis (Table S2).

### 2.6 Addressing communication gaps from sample collection to report generation

In the second year of the study, a mobile app was introduced with ethics approval to enhance real‐time communication on logistics and to support ongoing benefit–risk evaluation. While the app did not facilitate direct interaction with GPs, it allowed for the documentation of patient feedback on the sample collection and report delivery process. To reduce data fragmentation and improve diagnostic accuracy without increasing costs, access to previous pathology test results relevant to the analyzed genes was provided. This enabled the integration of genetic risk factors potentially triggered by environmental exposures with biochemical abnormalities in patient reports. Patient follow‐up was performed in August 2024, followed by recontact of the referring GPs detailing the most important findings for further discussion as part of continued professional development.

## 3 RESULTS

### 3.1 Patient enrolment into the pilot program

The strategy proposed for targeting multiple NCD pathways in health insurance beneficiaries (Figure 1) was approved after rigorous evaluation prior to implementation of the pilot program. Challenges encountered by the PFP included outdated practice contact details at the claims level and having to follow‐up on telephone calls despite promises that the practice will call back at a time that is suitable for the GP. In 28 of 65 cases (43%) where feedback could be obtained, the reasons for discontinuation of communication were related to medical scheme data, while 14 (22%) were related to issues at the medical practice, and 23 (35%) were due to attrition caused by patient resignation from the scheme, mostly due to employer changes and death. Three of the 135 doctors (2.2%) invited expressed immediate disinterest in the pilot and refused to consult their patients or obtain a sample for genetic testing. The reasons provided were:

1. “I am too old to do new things.”
2. “Our practice does not deliver that service to healthcare funder beneficiaries.”
3. “We are a cash practice and require confirmation of payment before the call will be transferred to the doctor.”

**FIGURE 2:**
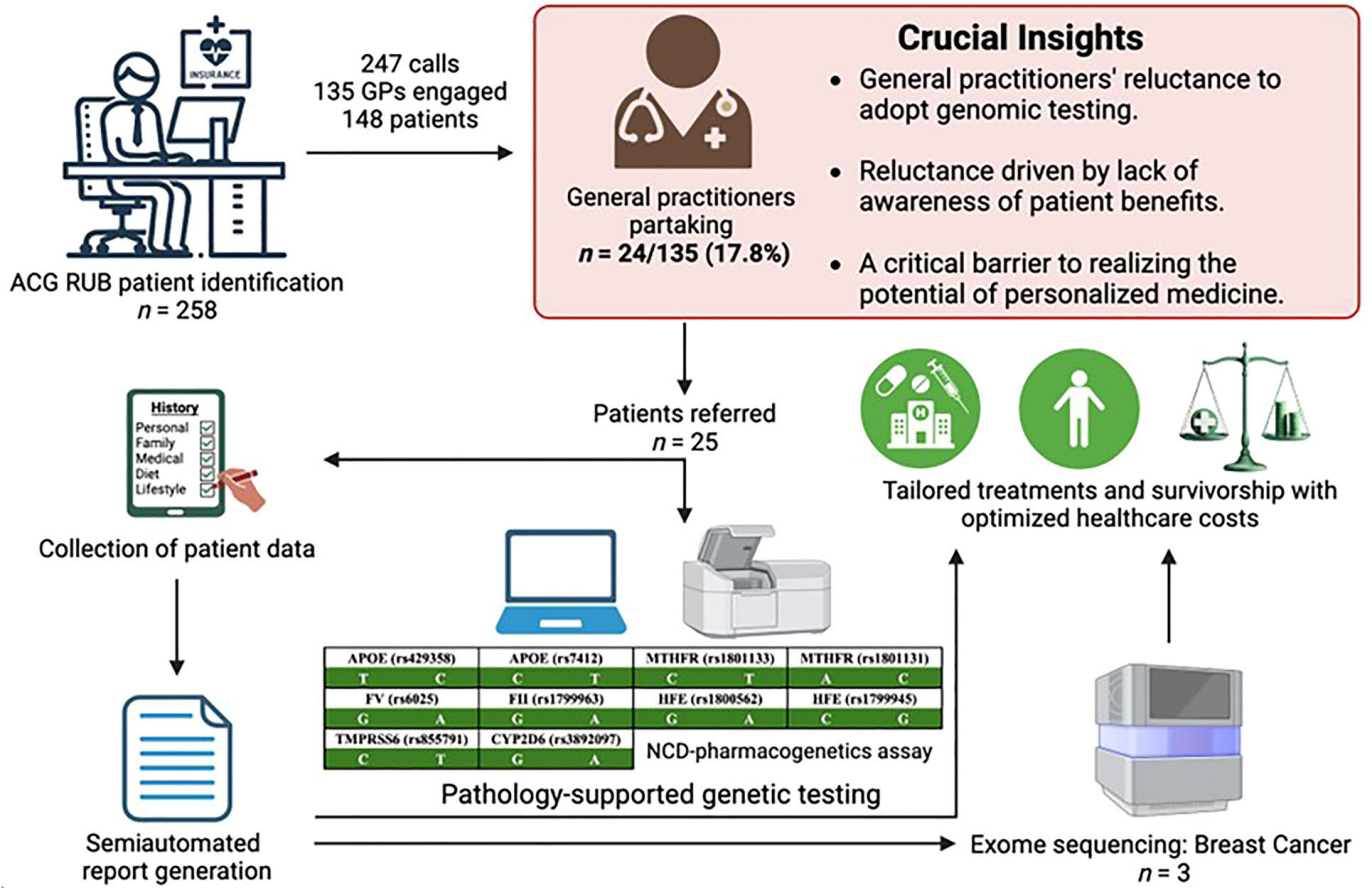
Enrollment of healthcare funder beneficiaries and first‐tier genetic testing after removing the cost barrier for patients, revealing critical insights to guide future reimbursement policy decisions. Image created with BioRender (https://biorender.com/). ACG RUB, Adjusted Clinical Groupers and Resource Utilization Band; GP, general practitioner; NCD, noncommunicable disease.

Although most doctors were comfortable speaking to the PFP and expressed appreciation for the medical scheme's vision by offering a “valuable program that is reimbursed” for genetic testing to improve patient care, mixed responses were obtained from group practices operating from different geographical areas. Figure 2 presents the results of enrolling healthcare funder beneficiaries into the pilot access program based on medical scheme claims data, together with the insights gained from this process reflecting the achievement of the first study objective. Of the 148 sample collection kits distributed to medical practitioners, 25 patient samples were returned for genetic testing. Patient reports were securely provided to the referring GPs via a website link for follow‐up consultations as part of the wellness and chronic disease screening components detailed in Table 1. The Gknowmix™ report algorithm structured the integrated clinical, pathology, and genetic data into a summary that facilitated the selection of three patients for WES. Lifestyle risk reduction guidelines were provided to all 25 patients based on the *APOE*‐dyslipidemia, *MTHFR*‐folate/homocysteine, *FII/FV*‐blood clotting, and *HFE*/*TMPRSS6* iron metabolism pathways. Given the dual role of *BRCA1/2* in HBOC and in influencing the response to poly (ADP‐ribose) polymerase (PARP) inhibitors due to their involvement in DNA repair mechanisms, familial risk and therapy‐associated risk were the most important clinical indicators supporting the extension of genetic testing to WES, beyond CYP2D6*4 included in the first‐tier NCD‐pharmacogenetics assay (Figure 2). The practical implications of the results were communicated to each patient by recommendations spanning across all three risk categories, including (1) encouraging the sharing of reports with family members who might benefit from familial risk information; (2) addressing metabolic syndrome features and genetic factors influencing biochemical abnormalities related to identified lifestyle risk; and (3) optimizing drug metabolism based on the need for a change in medication/dosage and the role of nutrient cofactors required for enzymatic function relevant to the gene variants analyzed. Despite the low response rate limiting reliable quantification of post‐test outcomes, exposing the reasons for an apparent reluctance among nonparticipating GPs to align with the principle of beneficence in personalized medicine, paved the way for a new medical scheme reimbursement model using PSGT.

### 3.2 First‐tier genotype screen extended to whole exome sequencing in eligible patients

The decision to perform WES was based on the age at diagnosis and family history of participants, as well as metabolic and biochemical abnormalities that could not be explained by the lifestyle and pharmacogenetic risk factors evaluated across six NCD pathways in this study (Table S1). A diagnosis of FH eligible for WES beyond *APOE*‐dyslipidemia was excluded based on standard clinical criteria including lipid profiles that were available for six patients. Ten patients tested positive for the *APOE* e4 allele, including two compound heterozygotes with the e2/e4 genotype, and one homozygote with the *APOE* e4/e4 genotype. International studies have demonstrated that the latter genotype is associated with an age‐ and sex‐dependent absolute 10‐year risk of approximately 25% for Alzheimer disease by the age of 80 years, studied across ethnic groups. The *APOE* e4 homozygote identified in our study is an African male diagnosed with depression in adolescence, who stopped the use of antidepressants shortly before enrollment in the pilot program at the age of 20 years. Since this patient was also homozygous for the *MTHFR* 677 T‐allele previously implicated in depression and medication side effects, we did not extend the first‐tier NCD pathway analysis to WES. Instead, we highlighted the detection of genetic risk factors for hypercholesterolemia and hyperhomocysteinemia that are influenced by environmental risk factors such as the diet and alcohol consumption, exemplified by inadequate physical activity in this patient. Since his dietary folate score was too low (<14), folate intake of 800 ug per day (double the standard recommendation) with proportional increase of vitamins B6, B12, and riboflavin was encouraged, while maintaining a normal weight to prevent synergistic effects of *APOE* e4 and homocysteine accumulation that may combine to cause detrimental effects on metabolism and cognition. The finding that 12 (48%) participants were obese (BMI ≥ 30 kg/m^2^) and 16 (64%) had a high waist circumference, underscored the interconnectedness of the metabolic syndrome with the broad spectrum of NCDs evaluated according to the study inclusion criteria.

The decision to perform comprehensive pharmacogenetic testing using WES was based on the question whether a change of medication or drug dosage was considered by the referring GPs, due to treatment failure or medication side effects. Neither were clinically indicated for two patients previously diagnosed with cancer found to be heterozygous for CYP2D6*4 (1846G>A). One of these patients initially diagnosed with pancreas cancer, developed prostate cancer 5 years later. This was followed by a pulmonary embolism previously treated with rivaroxaban, and metformin treatment for type II diabetes. In the second patient, a female who had melanoma which was surgically removed, multiple comorbidities were reported that are partly compatible with the detection of one copy of the *APOE* e4 allele and compound heterozygosity for *MTHFR* 677C>T and 1298A>C. These comorbidities included hypercholesterolemia, vitamin B12 deficiency, hypertension, depression, type II diabetes, hypothyroidism, and deep vein thrombosis reported on the study questionnaire as an adverse response from use of hormone replacement therapy (estradiol). Treatment of depression with venlafaxine metabolized by CYP2D6 was stopped by the patient after 5 years, and replaced by an antidepressant that does not have the same effect on *CYP2D6* activity.

MDD was the most common medical condition reported for evaluation in relation to the drug metabolizing CYP2D6 enzyme and adequacy of nutrient cofactors required for MTHFR function. The use of antidepressants was reported in 12 of the 15 affected patients at referral, of whom nine (36%) were homozygous or compound heterozygous for the two above‐mentioned *MTHFR* SNPs. The majority of these individuals (8/15, 89%) had a low folate score based on the questionnaire assessment completed at entry into the pilot. Six (67%) patients with these risk‐associated genotypes were diagnosed with anxiety and/or depression, and four (44%) reported being treated for hypertension. The medical scheme claims data also identified a young female currently aged 20 years who has struggled with anorexia and depression since the age of 13 years and has been in rehabilitation three times. During the pretest genetic counseling session, it was noted that she has no significant family history of depression, although anxiety was reported for both her mother and grandmother. The patient relapsed during the COVID‐19 lockdown, experiencing severe side effects from prescribed medication, including projectile vomiting, dizziness, and fainting. Given the complexity of this case, a second opinion was sought from a CP, who reviewed the patient's documented information, requested additional data on previous medication use, and approved the first‐tier genotyping report with specialist input at no extra cost to the family. The post‐test genetic counseling session helped the family to understand that the NCD pathway analysis was not intended to diagnose the medical condition, but could help optimize further treatment. Follow‐up to obtain feedback on her current health status indicated a change in treatment with reduced cost implications. Due to significant health improvements, this patient currently lives independently.

Deep vein thrombosis (three cases) treated with warfarin or rivaroxaban, pulmonary embolism (one case), and recurrent pregnancy loss (one case) were rare among the patients selected for the pilot study. While the metabolic syndrome was a common feature, none of these patients tested positive for prothrombotic variants in the FII and FV genes, indicating other causes for these conditions in the respective families as communicated in the patient reports. Nonalcoholic fatty liver disease as the hepatic manifestation of the metabolic syndrome was a prominent feature in a patient who previously experienced pregnancy loss and reported treatment with warfarin for pulmonary embolism during recent follow‐up. This patient expressed gratitude for gaining a better understanding of cumulative risk imposed by detection of *APOE* e4 heterozygosity in the presence of dyslipidemia and morbid obesity, as well as compound heterozygosity for *MTHFR* 677 C>T and 1298 A>C in the presence of a low folate score. These findings imply hyper‐responsiveness to biochemical abnormalities related to lipid and folate/vitamin B12/homocysteine metabolism, which resulted in recommendations for targeted lifestyle intervention extending to hypertension and depression also reported.

Coinheritance of both an iron‐loading *HFE* and iron‐lowering *TMPRSS6* variant detected in three patients highlighted the importance of combining serum iron studies with genetic testing, as environmental exposures such as regular blood donation or a vegetarian diet will determine the clinical relevance of this genotype; hence, we included enquiry about the latter two aspects in the study questionnaire. None of the study participants were homozygous or compound heterozygous for *HFE* H63D and C282Y, which are the most common cause of autosomal recessive HH. Carrier status for *HFE* C282Y detected in one patient has clinical implications for other family members, as the risk of transmitting two copies of the faulty gene to offspring is 25% when both parents are carriers. Early detection of a genetic predisposition for HH is crucial and requires knowledge of both transferrin saturation and ferritin levels for clinical interpretation, with ferritin levels above 1000 ng/mL in HFE‐related iron overload being associated with irreversible organ damage. Three females reported a medical history of iron deficiency in addition to other medical conditions, which were evaluated in relation to the metabolic syndrome and the *TMPRSS6* 2207 T‐allele for risk stratification according to lifestyle and inherited risk factors, aimed at mitigation of combined effects. Homozygosity for this allele was detected in one of these patients previously diagnosed with breast cancer, presenting with borderline low levels of ferritin (19 ng/mL), transferrin saturation (20.2%), and vitamin D (17 ng/mL). Although different laboratories vary in their classification of optimal vitamin D levels, blood levels above 35 ng/mL (and below 100 ng/mL) are generally considered desirable for cancer prevention and the alleviation of therapy‐induced side effects such as joint pain and osteoporosis. This was clinically relevant due to treatment with aromatase inhibitors, which are known to increase the risk of bone loss. In this context, the study RD highlighted the modifiable risk factors detected in relation to anthropometrical values outside the recommended reference ranges.

Genetic counseling support, available to all patients, was particularly helpful in managing expectations regarding familial inheritance, comorbidities, lifestyle, and pharmacogenetic risks for three breast cancer patients eligible for WES. This comprehensive analysis enabled the simultaneous assessment of breast cancer and associated comorbidities in a single test. Pathogenic variants in *BRCA1/2* and other high‐ to moderate‐risk cancer susceptibility genes were excluded based on WES in all three cases, diagnosed with breast cancer at the ages of 28 (Case 1), 45 (Case 2), and 63 (Case 3). The study GC summarized the WES results in follow‐up letters to the referring doctors, highlighting recommendations based on familial risk and lifestyle factors, which were quantified and incorporated into patient reports from the study questionnaire. The identification of modifiable lifestyle factors that influence the effects of low‐penetrance gene variants evaluated in the first‐tier genotyping process not only aided in selecting patients for extended genetic testing, but also facilitated the interpretation of WES results. Follow‐up of Case 1 and Case 2 after 3 years resulted in updating of the health monitoring section of the patient reports, while Case 3 was lost to follow‐up.

Given that Case 1 was diagnosed with breast cancer at a very young age and presented with comorbid metabolic syndrome, anxiety, and depression, WES was deemed appropriate. This decision was justified based on the Manchester Scoring system predicting a more than 20% chance of a *BRCA1/2* pathogenic variant in the family, a 13% CanRisk prediction for a causative moderate– to high‐penetrance gene variant, and a moderate lifetime risk for contralateral breast cancer. However, despite increasing the number of cancer susceptibility genes analyzed in the WES data from the initial 28 genes to more than 100 in the research arm of the pilot, we could not identify a *BRCA*/other high‐penetrance pathogenic variant responsible for the early onset HBOC in the family. The family history included colorectal and prostate cancer in the father between the ages of 40 and 50 years, ovarian cancer in a cousin aged 30 years, and unspecified cancers in her uncle and mother between 50 and 70 years. A rare variant of uncertain clinical significance with conflicting evidence of pathogenicity was detected in the moderate‐risk gene *PMS2* (rs1805323), together with several nonsynonymous SNPs in low‐penetrance genes associated with metabolic and cancer risk (data not shown). This raised the possibility of polygenic inheritance in a high‐risk environment, as reflected by type II diabetes treated with metformin and glibenclamide, marking a diagnosis of the metabolic syndrome in the presence of comorbid hypertension and central obesity. In response to the question whether the patient had known nutrient deficiencies, the GP noted administration of vitamin B12 injections. This was supported by detection of genetic variation in the one‐carbon metabolism pathway and a low dietary folate score of 2, which should ideally be above 13 due to inverse association with homocysteine levels, which in turn correlates with high BMI and depression relevant to this patient.

**FIGURE 3:**
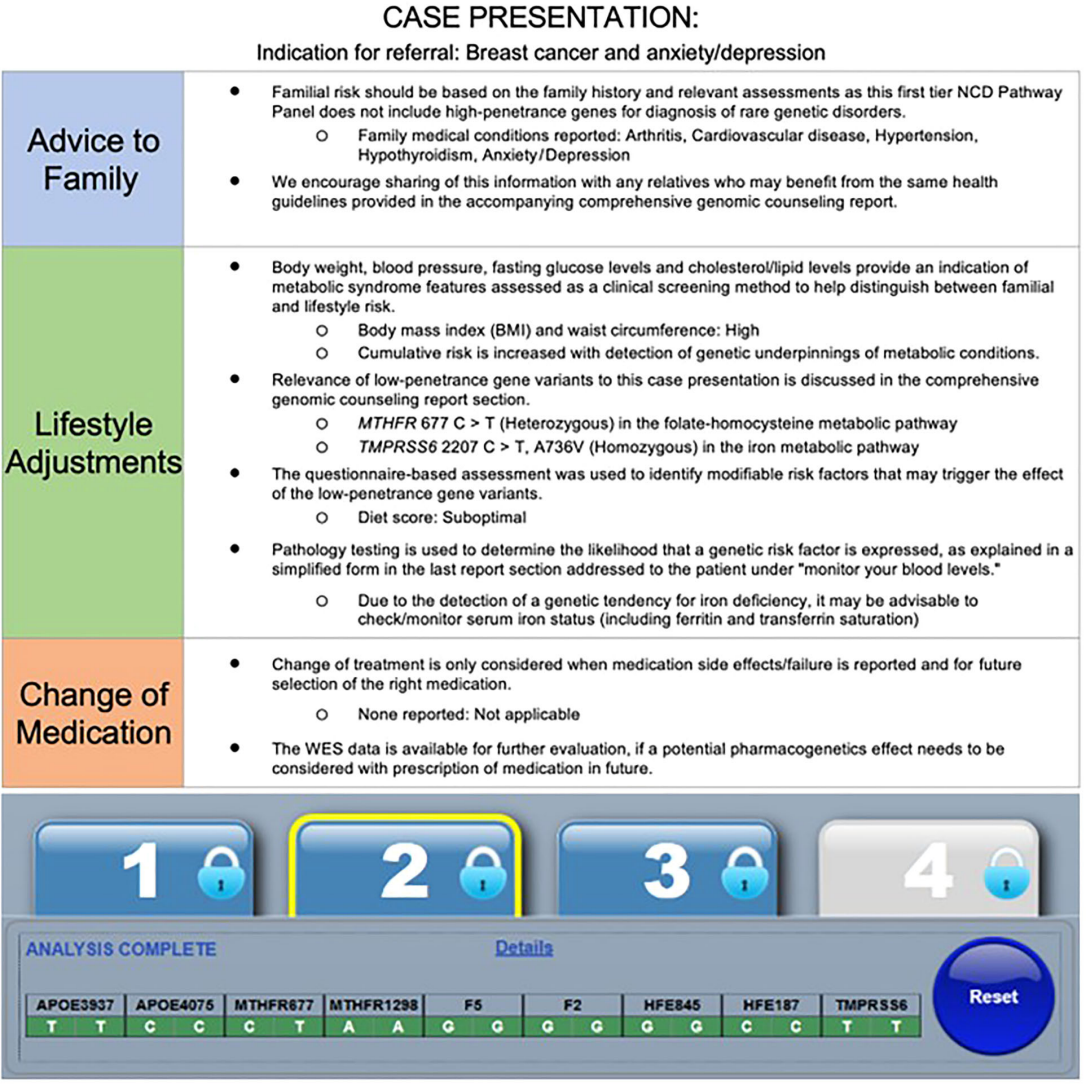
Excerpt from a pathology‐supported genetic testing report of multiple noncommunicable disease (NCD) pathways influenced by familial risk and environmental factors. Top: Multidisciplinary risk stratification summary extracted from the comprehensive genomic counseling and health monitoring sections of the NCD point‐of‐care assay extended to whole exome sequencing (WES). Bottom: Detection of two low‐penetrance gene variants associated with homocysteine accumulation (*MTHFR* 677 CT heterozygote) and a genetic tendency for iron deficiency (*TMPRSS6* 2207 TT homozygote), respectively.

The first‐tier genotype results of Case 2 are shown in Figure 3, within the clinical context used to determine eligibility for WES and to facilitate the differential diagnosis of inherited versus lifestyle‐related breast cancer in this patient. Although no family history of cancer was reported, WES was deemed appropriate based on international guidelines that recommend NGS for patients diagnosed with breast cancer under the age of 50 years. In line with the small chance for a causative mutation based on the Manchester (<10%) and CanRisk (6%) scoring systems, we could not find a single‐gene cause for her breast cancer. This patient who was diagnosed with ductal carcinoma in situ and treated with radiation only, also reported MDD from the age of 20 years, treated effectively with bupropion and trazodone as indicated by the referring GP on the study questionnaire. This is in line with the fact that there are currently no established recommendations for genetically guided selection or dosing of trazodone metabolized by *CYP3A4*, while potential drug–drug interaction between and CYP2B6 inducers or inhibitors was not relevant to this case. The two low‐penetrance gene variants detected in the *MTHFR* gene (677 C>T, heterozygous) and the *TMPRSS6* gene (2207 C>T, homozygous) may at least in part explain the fatigue and medical history of depression reported. The *MTHFR* SNP may reduce DNA methylation in cancer patients and this effect should be interpreted in relation to serum folate, vitamin B12, and homocysteine levels. No action should be taken based on detection of the iron‐lowering *TMPRSS6* and/or iron‐loading *HFE* missense mutations when *s*erum iron parameters are within the normal ranges. This patient was pleased that the GC discussed so many aspects of her cancer diagnosis, from considering the role of environmental and genetic causal factors to treatment for anxiety and depression, in addition to revisiting her family history for heart disease in her paternal line and depression on her maternal side. This information was important for interpretation of the first‐tier genotyping assay in the context of relevant biochemistry levels and WES performed as the next step. Upon follow‐up in 2024 when the risk stratification approach refined during the study as presented in Figure 1 was demonstrated to the patient, she raised the concern that her breast cancer may have been triggered by infertility treatment. Although previous studies suggest a slight increase in breast cancer risk shortly after treatment, long‐term risk does not appear to be significantly elevated. Current use of fluoxetine was prescribed by her psychiatrist, indicating the importance of a multidisciplinary approach and follow‐up reports enabled by our adaptive reporting system on request.

### 3.3 Feedback on the process and outcomes

Follow‐up was performed to assess whether (1) all the patients received their reports; (2) the report was of benefit to the patients, and (3) whether they have any complaints, concerns, or questions. Two of the first three responses answered “Yes” to the first two questions and “No” to the third question. One of these patients included the following statement “The Doctor [GC] was such a help, explained everything to me as well and she was very patient. Thank you, guys, for the great service.” Since the remaining patient responded, “I have not received the report so I am still in the dark and I would love to know the feedback of the test,” her report was updated after further discussion and shared with her new GP. Both patients appreciated the follow‐up meetings and expressed concerns about lack of awareness among GPs of the patient benefit that could be derived from genetic testing using a three‐pronged approach for simultaneous analysis of inherited, lifestyle‐trigged, and therapy‐associated pathologies. This is evidenced by the following comment from a doctor contacted about delivery of the patient report by the care coach appointed by the medical scheme: “We have treatment algorithms and will get to the right treatment eventually.”

**FIGURE 4:**
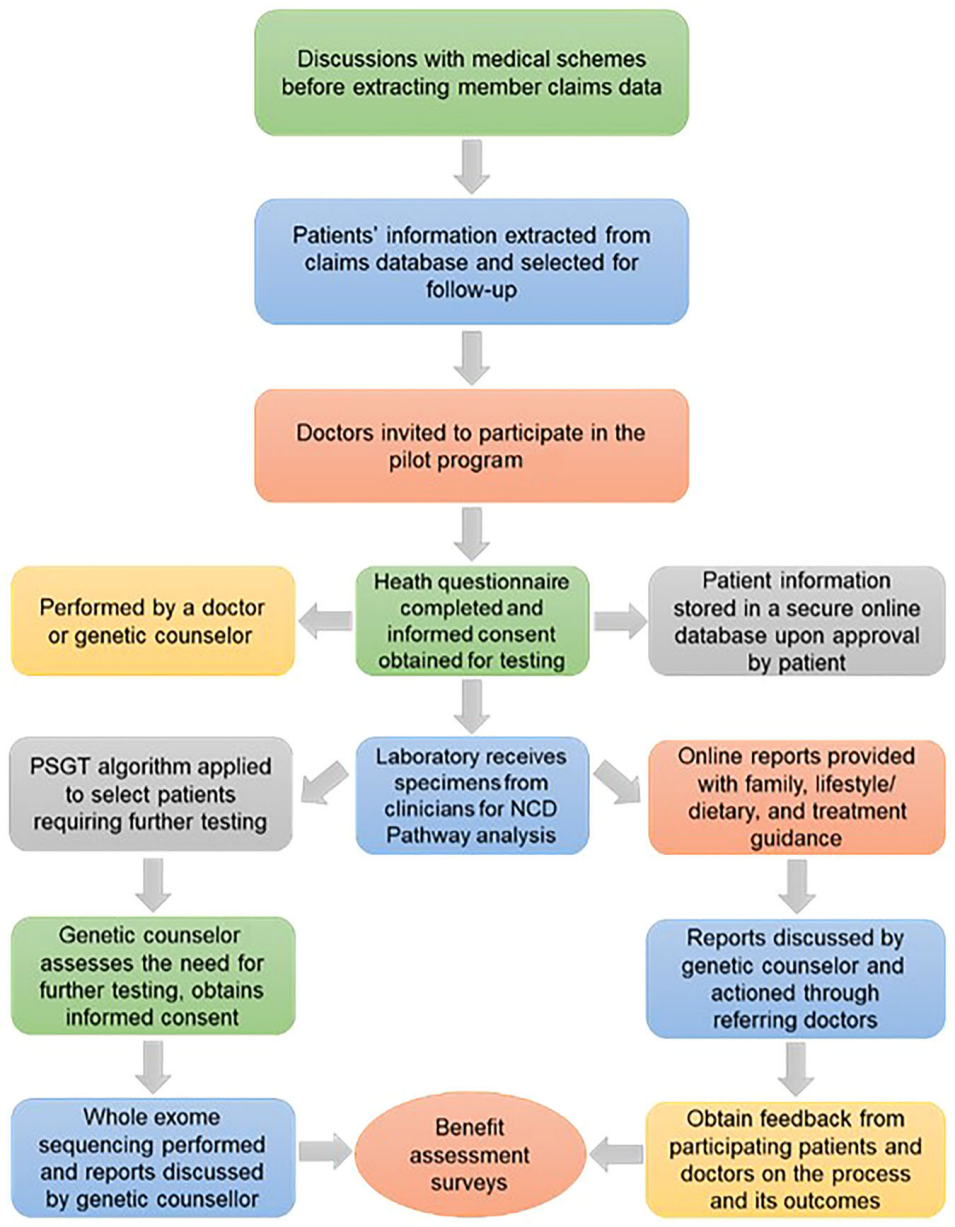
Open Genome Project workflow refined during this study as a new medical scheme reimbursement model for noncommunicable diseases (NCDs) with an intervening genetic component. This process enables data sharing among healthcare professionals for adaptive reporting and participation of patients through wellness surveys during follow‐up, for application of personalized medicine using an integrated service and research approach. PSGT, pathology‐supported genetic testing.

Although patient follow‐up is ongoing, those contacted to date expressed appreciation for the nutrition information provided by the RD and counseling support by the GC. A particularly positive response came from a postmenopausal woman with a previous diagnosis of hormone receptor‐positive breast cancer. She suffered severe chemotherapy side effects, which resulted in permanent peripheral neuropathy. Her GP advised her to contact the research team for further clarification of the genetic results, leading to post‐test genetic counseling by the GC. As a result of the NCD pathways report, she increased her exercise level and adjusted her diet, appreciating its “clear and gentle guidance in areas highlighted for improvement.” Patient experiences’ contributed to optimization of the workflow for our new medical scheme reimbursement model for NCDs, as outlined in Figure 4.

## 4 DISCUSSION

Comprehensive genomic sequencing using WES was introduced for the first time in GP practice, supported by genetic counseling and first‐tier genotyping, and informed by a wellness survey (study questionnaire) completed by 25 health insurance beneficiaries. The major challenge experienced was that the majority of the doctors (80%) who initially agreed to participate did not use the saliva sample collection kits provided for the pilot. This expense of unused kits that has since expired was increased by high courier costs of sending samples to sometimes remote areas in South Africa. Some group practices operate from different geographical spaces, with some doctors who have agreed to participate and others who refused to refer patients for genomic testing. Emergence of COVID‐19 a few months after initiation of the study may have contributed to these mixed responses. This hampered the implementation of the pilot program, which was further challenged by the difficulty experienced by some patients to obtain their reports and an explanation of the findings by their GPs. While our primary objective of enrolling healthcare funder beneficiaries into the pilot program was successfully achieved, the low uptake of 17.8% suggests a potential misunderstanding of the clinical and ethical implications of declining opportunities designed to enhance healthcare.

The GC performed a qualitative study using the theoretical framework approach for data analysis prior to this pilot study, which identified significant barriers to WES implementation in South Africa (Van Der Merwe et al., 2022). Recommendations to overcome these barriers included securing funding and buy‐in from both the private and public sectors, as well as from medical insurance companies, which was successfully accomplished in this study. WES was offered at no cost to selected patients as part of postgraduate student training, initiated by first‐tier genotyping to help educate clinicians about their role in broading access to genomic medicine.

The translation of genetic research into clinical practice typically begins by focusing on specific health concerns or disorders and validating relevant assays within the clinical context where the service will be utilized (Cleeren et al., 2011). In this study, the likelihood of the metabolic syndrome was first assessed as a unifying risk factor for NCDs, within predefined inclusion criteria focused on medical conditions studied extensively in South Africa over more than three decades. The rationale for this approach has previously been described by Kotze et al. (2015) to facilitate differential diagnosis of inherited versus lifestyle‐triggered NCD subtypes. Identifying a genetic predisposition may have implications for other family members, therefore the limited availability of registered GCs trained in the diverse genomic applications offered by WES necessitated the development of a new reimbursement model. During this process, we adhered to genetic principles aimed at minimizing the risk of genetic discrimination, as previously discussed in relation to health insurance (Kotze et al., 2013). Since the SNPs selected for first‐tier genotyping are common in the general population (>1%), transparent communication of all known interacting factors in patient reports and correlation with relevant biochemical parameters are crucial as facilitators of uptake within an ethical framework. The data are stored for future reference, should it be needed, based on the patients' health status and the informed consent provided at referral. Reinterpretation of the variant of uncertain clinical significance detected by WES in Case 1 diagnosed with the metabolic syndrome, may become relevant as new information emerges in the literature, and the genetic analysis may in future also be extended to whole genome sequencing as the most comprehensive genomic assay available in South Africa. Failure to detect a pathogenic cancer susceptibility variant in Case 2 substantiated the value of including the assessment of the metabolic syndrome in risk stratification to provide health guidelines beyond the identification or exclusion of a single gene using targeted NGS or WES (Okunola et al., 2023).

Detection of variation in the *APOE* gene in 13 (52%) of our study participants provides a practical example of the benefit of diet and lifestyle intervention as the treatment of choice, without the need for lipid‐lowering medication in dyslipidemics prior to the onset of CVD (Kotze et al., 2013). The clinical dilemma of statin overtreatment in patients without FH could be addressed effectively by early detection of the *APOE* e4 allele using the PSGT approach, incorporating WES preceded by first‐tier genotyping regardless of population/ethnic group (Marais et al., 2019). We have previously demonstrated that the *APOE* e4 allele is associated with raised total and low‐density lipoprotein (LDL) cholesterol levels, but to a lesser degree compared to FH. It is unethical to administer chronic medication or chemotherapy in patients who are unlikely to benefit (Kotze et al., 2024). The *APOE* e4 allele poses significant risk for late‐onset Alzheimer's disease, which may be mitigated by addressing modifiable risk factors associated with raised lipid levels (Livingston et al., 2024). The finding that lifestyle modifications are beneficial at any age underscores the urgency for risk reduction intervention in South African patients at increased risk of heart disease and associated comorbidities (Kotze & Van Rensburg, 2012; Lückhoff et al., 2016). We used the Framingham risk score (FRS) as a clinical tool for determining the 10‐year CVD risk, heart age, and the need for statin treatment in six individuals (five females and one male) in the study cohort with available lipid levels. *APOE* e4 heterozygosity was detected in three of these six patients. Based on age, gender, blood pressure, cholesterol levels (total and HDL‐cholesterol), hypertension treatment, smoking, and diabetes status, four beneficiaries were calculated to have a low 10‐year risk of CVD (<10% and <11.2% risk for females and males, respectively). One female treated for diabetes had a moderate risk of 15.9%, while a male participant with a high risk for CVD of 29.4% was treated with a cholesterol‐lowering statin. In patients with high triglyceride levels in the presence of the *APOE* e2 allele detected in three cases, obesity, diabetes, or hypothyroidism where highlighted if present, representing a second hit for the development of the genetic disorder hyperlipoproteinemia type III. Although there was no clinical indication for a change of medication in the dyslipidemia group, patients could still benefit from the lifestyle changes highlighted by the RD using an experimental approach subject to clinical monitoring.

Currently, efforts to prevent complex diseases through lifestyle modification are not universally achieved, highlighting the need for whole‐of‐life healthcare approaches using public health genomics tools designed to facilitate risk classification and early intervention (Bauer et al., 2014; Cesuroglu et al., 2009; Van Der Poort et al., 2024). Obesity was identified as a major risk factor in our study participants that may be related to the relatively high *MTHFR* variant allele frequency. Previous findings in other populations demonstrated the combined effect of *MTHFR* 677 C>T and low folate status on BMI and homocysteine levels, which were replicated in South African patients with depression (Delport et al., 2014), multiple sclerosis (Davis et al., 2014), and postmenopausal hormone‐positive breast cancer (Baatjes et al., 2023). PSGT enables simultaneous implementation of both a top‐down (phenotype‐to‐genotype) and bottom‐up (genotype‐to‐phenotype) approach, interconnected by intermediary phenotypes caused by gene–environment interaction. The value of this approach was exemplified by Pretorius et al. (2022), who reported a high prevalence of metabolic syndrome features such as dyslipidemia, hypertension, and type 2 diabetes mellitus among South African patients suffering from post‐acute sequela of coronavirus 2019 (PASC)/Long COVID. Similar to the findings of Purcell et al. (2021) attempting to implement a Whole Health System of Care “when COVID happened,” our combined chronic disease and wellness program may be leveraged to support those most at risk after the COVID‐19 pandemic.

Interpretation of the genetic results in the context of each patient's clinical characteristics followed a different reporting format than that described by Hao et al. (2022), which illustrates the boundary where the role of the clinical laboratory ends and the role of the treating physician begins. The NCD pathways reports extended to WES in three patients uniquely provide clinical and lifestyle guidance aligned with biochemistry test results reported as “not provided,” “normal,” “high,” or “low.” These categorizations indicate whether the genetic results are diagnostic or predictive in the presence or absence of relevant environmental factors also evaluated in each patient and included in the reports. The benefits of NCD pathway analysis elevated to WES in the context of familial risk include the ability to evaluate an unlimited number of genes associated with the diagnosis, increasing the chances of identifying the genetic cause of the disease in the family, with subsequent predictive testing and management options (prophylactic surgery, screening, and surveillance) for at‐risk family members (Van Der Merwe et al., 2017). With regards to comorbidities, evidence‐based lifestyle recommendations were provided in conjunction with a monitoring plan that includes biochemical testing of pathways that require optimization. For assessment of treatment‐related risk, sequencing of entire drug metabolism genes may point to the reason for reduced drug efficacy/failure or side effects and enable consideration of alternative treatment options by the patient's treating physician.

Our study was limited by the small number of participants and underutilization of pathology in guiding risk reduction strategies across various disease entities. Previous pathology test results are crucial to determine the clinical relevance of genetic variants (Kotze et al., 2015, 2013; Van Der Merwe et al., 2024). Generation of contextualized patient reports nonetheless proved effective to determine whether NCDs associated with biochemical abnormalities or medication side effects/failure may be caused by genetic or environmental risk factors, or both. The referring GPs rarely indicated a need to change medication as patients may also be treated by other specialists not involved in the pilot, which was identified as another limitation that needs to be addressed in future. The Open Genome Project aims to facilitate benefit‐sharing within an evolving interdisciplinary workflow as new data sources are incorporated into the piloting platform. This project contributed to a better understanding of the processes and needs of the health sector, reflecting a new normal as reported by Wolf and Green (2023) in the context of clinical frontline research and patient interaction. Ultimately, the joint analysis of outcomes and costs using real‐life examples will enable wholeness in healthcare that drives desired outcomes and delivers value across the illness and wellness domains (Levins, 2014).

### 4.1 Future directions

Many previous studies have emphasized the need to integrate genomic medicine into the management of common chronic adult diseases, an opportunity explored in our pilot medical scheme program. Acknowledgment of PSGT as best practice in precision oncology by the International Consortium of Personalised Medicine (ICPerMed) in 2020 marks a long and challenging path involving intensive clinician and scientist education toward the incorporation of genetic knowledge into the workflow of busy medical practices. Our research translation protocol includes transferring of individual laboratory‐based genotyping assays to a portable instrument, using a novel multiplex test kit to ensure patient entry into the right care pathway before advancing to costly NGS technologies. The cost‐saving potential of POC testing is the focus of a second pilot program recently conducted as an extension of the current study. Scheduled meetings requested by individual group practices based on their experiences with PSGT are the next step toward implementing WES through the six metabolic pathways assessed in this study.

Our focus on genetic counseling and family involvement in a combined chronic disease and wellness program offers crucial support in managing patient expectations and genetic risks associated with germline DNA testing. Although the value of genetic counseling as part of a patient‐centered genetic testing process is clear, only a few medical schemes in South Africa currently reimburse counseling consultations, typically after patients have paid out of pocket. This must change to facilitate the broader adoption of our medical scheme reimbursement model that supports the use of POC testing for integration of germline and biochemical/tumor genetics as a new vision for the future of translational pharmacogenomics (Mampunye et al., 2021; Okunola et al., 2023). The recent reimbursement of tumor‐based NGS, following NCD pathways analysis, by the participating medical scheme marks significant progress in this direction. PSGT acknowledges the negative impact of folate deficiency in patients with homologous recombination deficiency treated with PARP inhibitors, such as Olaparib.

## 5 CONCLUSION

The successful integration of PSGT within a dynamic interdisciplinary workflow was demonstrated for the first time in patients with a history of frequent clinic visits. Contrary to general assumptions, the cost of genetic services was not the primary barrier. Instead, the main challenge was the reluctance of most GPs to adopt genomic testing, likely due to a lack of awareness about its benefits when aligned with pathology test results to prevent harmful, unnecessary interventions based solely on genetic test results. During follow‐up, some patients reported prolonged symptoms following COVID‐19 infection (data not shown), which may have contributed to the limited patient enrolment in the pilot. In preparing patient reports and recent updates where relevant, priority was given to reviewing previous test results as part of the data collected for analysis. Clarifying the meaning of detected genetic variants, in the presence or absence of known environmental factors, is essential to explaining or predicting biochemical abnormalities associated with NCDs. Therefore, assessment of the relevant biochemistry or histopathology is crucial to facilitate clinical interpretation. Due to the three‐pronged approach applicable to both the first‐tier NCD pathways analysis and WES, all patients received actionable results irrespective of the detection of a pathogenic variant as the usual focus using targeted NGS.

We believe this pilot program effectively assessed and maximized stakeholder benefits, particularly for medical scheme members, by increasing access to testing and personalized risk reduction recommendations. For healthcare funders, the increased initial expenditure would translate into improved health management and reduced long‐term medical costs. Genetic testing and counseling service providers stand to benefit from medical scheme reimbursement, which can drive increased uptake while supporting GPs in implementing effective treatment plans informed by a better understanding of multifactorial diseases with a genetic component. As more clinicians recognize the value of precision or personalized genomic medicine in reducing healthcare costs associated with NCDs, similar genetics access programs may be implemented in the future. The weight of current scientific evidence in relation to risk–benefit assessment supports our novel genotyping approach with proven relevance to several clinical domains.

## AUTHOR CONTRIBUTIONS

Study design: Manie De Klerk, Lindiwe Whati, and Maritha J. Kotze. Process refinement: Johny Erasmus, Lindiwe Whati, and Maritha J. Kotze. Data collection: Manie De Klerk, Nicole Van Der Merwe, and Maritha J. Kotze. Contribution to innovative tools and illustrations: Nicole Van Der Merwe, Johny Erasmus, Daniel W. Olivier, and Maritha J. Kotze. Data analysis: Manie De Klerk, Nicole Van Der Merwe, Lindiwe Whati, Kelebogile E. Moremi, and Maritha J. Kotze. Manuscript preparation, editing and final approval for publication: All authors.

## CONFLICT OF INTEREST STATEMENT

Manie De Klerk (part‐time contracted medical advisor) and Johny Erasmus (full‐time case manager) were employed by the managed care company that rendered the services to the medical scheme during the period of this project. Nicole Van Der Merwe (medical scientist and genetic counselor [GC]) was employed part‐time by the University of Stellenbosch and subsequently established the telegenetics company, FamGen Counselling Pty Ltd. Lindiwe Whati is a shareholder and Daniel W. Olivier is a consultant for Gknowmix Pty Ltd. Maritha J. Kotze is the founder director and a shareholder of Gknowmix Pty Ltd. to enable the integration of research and service delivery as part of a scientist training and clinician education program. The other authors declare no conflicts of interest.

## PATIENT CONSENT STATEMENT

All patients provided informed consent for research participation.

## PERMISSION TO REPRODUCE MATERIAL FROM OTHER SOURCES

Figure 1 was reproduced with minor modification from the white paper Accelerating Excellence in Science in Africa (AESA) (2021), following personal communication with Prof. Nicola Mulder (November 7, 2023). Permission for use of the material provided in Figure 3 was obtained from the corresponding author, as a founder director of Gknowmix Pty Ltd.

## Supporting information




**TABLE S1** Whole exome sequencing prescreen conducted in healthcare funder beneficiaries based on the detection of clinical characteristics and lifestyle factors relevant to the first‐tier genotype data incorporated in a unique NCD pathways report for each patient.
**TABLE S2** Cancer susceptibility genes included in the virtual whole exome sequencing panel for three breast cancer patients.

## Data Availability

Data not already included in the manuscript will be made available on request within the constraints of the ethical requirements for the Open Genome Project.
