# Supplementary material for: Incorporating familial risk, lifestyle factors, and pharmacogenomic insights into personalized noncommunicable disease (NCD) reports for healthcare funder beneficiaries participating in the Open Genome Project
Source: Ann Hum Genet. 2024 Oct 29;89(4):208–27. doi: 10.1111/ahg.12582 (PMC12152532; doi:10.1111/ahg.12582)
Supplement: Supplementary file 1 — TABLE S1 Whole exome sequencing prescreen conducted in healthcare funder beneficiaries based on the detection of clinical characteristics and lifestyle factors relevant to the first‐tier genotype data incorporated in a unique NCD pathways report for each patient. TABLE S2 Cancer susceptibility genes included in the virtual whole exome sequencing panel for three breast cancer patients. [file AHG-89-208-s001.docx]

**Supplementary Table S1**. Whole exome sequencing pre-screen conducted in healthcare funder beneficiaries based on the detection of clinical characteristics and lifestyle factors relevant to the first-tier genotype data incorporated in a unique NCD pathways report for each patient.

| **Variables, n** | **Female** | **Male** | **Total** |
| --- | --- | --- | --- |
| **Diagnosis: Inclusion criteria^†^** | 22 | 3 | 25 |
| Age, years (Mean ± SD) | 48.7 ± 20.6 | 47.3 ± 24.0 | 48.5 ± 20.5 |
| Hypercholesterolaemia | 4 | 0 | 4 |
| Cancer | 7 | 1 | 8 |
| Depression | 13 | 2 | 15 |
| Deep vein thrombosis | 3 | 0 | 3 |
| Pulmonary embolism | 1 | 0 | 1 |
| Recurrent pregnancy loss | 1 | - | 1 |
| Iron deficiency | 3 | 0 | 3 |
| **Metabolic syndrome features^‡^** |  |  |  |
| Waist Circumference | 14 | 2 | 16 |
| Glucose intolerance/ type II diabetes | 4 | 1 | 5 |
| Hypertension | 9 | 1 | 10 |
| Low HDL-cholesterol | 3 | 1 | 4 |
| High triglycerides | 1 | 1 | 1 |
| **Lifestyle** |  |  |  |
| Body mass index (kg/m^2^, Mean ± SD) | 31.1 ± 11.1 | 26.3 ± 8.1 | 30.5 ± 10.7 |
| 18.5 – 24.9 kg/m^2^ – normal | 9 | 2 | 11 |
| 25 – 29.9 kg/m^2^ – overweight | 2 | 0 | 2 |
| ≥30 kg/m^2^ – obese | 11 | 1 | 12 |
| Current Smoker | 3 | 1 | 4 |
| Alcohol Intake | 10 | 0 | 10 |
| Abstain | 1 | 0 | 1 |
| Occasionally, 1-3 units per week – low | 8 | 0 | 8 |
| 3–13 units per week – moderate | 2 | 0 | 2 |
| 14 or more units per week – high | 0 | 0 | 0 |
| Physical Activity (exercise per week) | 8 | 1 | 9 |
| High ( ≥4 times/intense daytime activity) | 0 | 0 | 0 |
| Moderate (2-3 times, sedentary) | 8 | 1 | 9 |
| Low (none/1 times week, sedentary) | 14 | 1 | 15 |
| Nutrition |  |  |  |
| Fat score |  |  |  |
| High (>26) | 4 | 0 | 4 |
| Moderate (22-26) | 2 | 1 | 3 |
| Low (16-21) | 7 | 2 | 10 |
| Very low (<16) | 8 | 0 | 8 |
| Folate score (Mean ± SD) |  |  |  |
| Very low (<6) | 13 | 1 | 14 |
| Low (6-10) | 4 | 2 | 6 |
| Moderate (11-13) | 1 | 0 | 1 |
| High (>13) | 4 | 0 | 4 |
| Fruit, vegetable and fibre score |  |  |  |
| High (>18, and/or ≥5 portions per day) | 0 | 0 | 0 |
| Moderate (14-18) | 4 | 3 | 7 |
| Low (7-13) | 11 | 0 | 11 |
| Very low (<7) | 7 | 0 | 7 |
| **Genotypes** |  |  |  |
| *Apo E* 4075 C> T, allele e2 (rs7412) |  |  |  |
| Not detected | 19 | 3 | 22 |
| Heterozygous | 3 | 0 | 3 |
| Homozygous | 0 | 0 | 0 |
| *Apo E* 3937 T> C, allele e4 (rs429358) |  |  |  |
| Not detected | 13 | 2 | 15 |
| Heterozygous | 9 | 0 | 9 |
| Homozygous | 0 | 1 | 1 |
| *MTHFR* 677 C > T, A222V (rs1801133) |  |  |  |
| CC - Not detected | 11 | 1 | 12 |
| CT – Heterozygous | 11 | 1 | 12 |
| TT – Homozygous | 0 | 1 | 1 |
| *MTHFR* **1298 A> C, E429A** (rs1801131) |  |  |  |
| AA - Not detected | 11 | 3 | 14 |
| AC – Heterozygous | 10 | 0 | 10 |
| CC – Homozygous | 1 | 0 | 1 |
| *FII* 20210 G > A (rs1799963) |  |  |  |
| GG - Not detected | 22 | 3 | 25 |
| GA – Heterozygous | 0 | 0 | 0 |
| AA – Homozygous | 0 | 0 | 0 |
| *FV* 1691 G > A, Leiden (rs6025) |  |  |  |
| GG - Not detected | 22 | 3 | 25 |
| GA – Heterozygous | 0 | 0 | 0 |
| AA – Homozygous | 0 | 0 | 0 |
| *HFE* 187 C>G, H63D (rs1799945) |  |  |  |
| CC - Not detected | 17 | 2 | 19 |
| CG – Heterozygous | 5 | 1 | 6 |
| GG - Homozygous | 0 | 0 | 0 |
| *HFE* 845 G > A, C282Y (rs1800562) |  |  |  |
| GG - Not detected | 22 | 2 | 24 |
| GA – Heterozygous | 0 | 1 | 1 |
| AA – Homozygous | 0 | 0 | 0 |
| *TMPRSS6* **2207 C>T, A736V (**rs855791) |  |  |  |
| CC - Not detected | 12 | 3 | 15 |
| CT – Heterozygous | 7 | 0 | 7 |
| TT – Homozygous | 3 | 0 | 3 |
| *CYP2D6* 1846 G>A, allele 4 (rs3892097) |  |  |  |
| GG - Not detected | 21 | 2 | 23 |
| GA - Heterozygous | 1 | 1 | 2 |
| AA - Homozygous (poor metabolizer) | 0 | 0 | 0 |
| **Available biochemistry** |  |  |  |
| Total-, HDL-, LDL-cholesterol, triglycerides | 5 | 1 | 6 |
| Vitamin B12, Serum folate, homocysteine | 0 | 0 | 0 |
| Transferrin saturation, ferritin | 3 | 0 | 0 |
| Glucose, HbA1c | 5 | 1 | 6 |
| Vitamin D | 2 | 0 | 2 |

**^†^**Some individuals had more than one medical condition. **^‡^**Metabolic syndrome characterized by three or more features listed represents a unifying risk factor incorporated into the report-generating algorithm to facilitate selection of uninformative cases for extended testing.

Abbreviations: SD = Standard deviation*, APOE,* apolipoprotein E; *FII,* factor V/prothrombin; *FV,* factor V; *HFE,* hereditary hemochromatosis gene; *MTHFR,* Methylenetetrahydrofolate reductase; *TMPRSS6,* Transmembrane protease, serine 6; *CYP2D6,* Cytochrome P450 family 2D member 6.

**Supplementary Table S2.** Cancer susceptibility genes included in the virtual whole exome sequencing panel for three breast cancer patients.

| **High** **penetrance** | **Moderate penetrance** | **Variable penetrance** | | |
| --- | --- | --- | --- | --- |
| BRCA1 NM_007294.3 | ATM NM_000051.3 | BARD1 NM_000465.3 | MSH6 NM_000179.2 | RAD51B NM_002877.5 |
| BRCA2 NM_000059.3.1 | BRIP1 NM_032043.2 | EPCAM NM_002354.2 | NBN NM_001024688.2 | RAD51C NM_002876.3 |
| CDH1 NM_001317184.1 | CHEK2 NM_007194.3 | FANCC NM_000136.2 | NF1 NM_001042492.2 | RAD51D NM_002878.3 |
| PTEN NM_000314.6 | PALB2 NM_024675.3 | MLH1 NM_000249.3 | PMS1 NM_000534.4 | RECQL NM_002907.3 |
| STK11 NM_000455.4 |  | MRE11A NM_005590.3 | PMS2 NM_000535.6 | MUTYH NM_001048171.1 |
| TP53 NM_000546.5 |  | MSH2 NM_000251.2 | RAD50 NM_005732.3 | XRCC2 NM_005431.1 |

Risk categories related to gene/mutation penetrance may differ between studies reported in the literature. Some genes are clinically relevant to both cancer predisposition and treatment targets.
